# Toxicology of pharmaceutical and nutritional longevity compounds

**DOI:** 10.1017/erm.2023.18

**Published:** 2023-06-22

**Authors:** Cigdem Kahraman, Duygu Kaya Bilecenoglu, Suna Sabuncuoglu, Irem Tatli Cankaya

**Affiliations:** 1 Department of Pharmacognosy, Hacettepe University Faculty of Pharmacy, Ankara, Turkey; 2 Faculty of Health Sciences, Aydın Adnan Menderes University, Aydın, Turkey; 3 Department of Pharmaceutical Toxicology, Hacettepe University Faculty of Pharmacy, Ankara, Turkey; 4 Department of Pharmaceutical Botany, Hacettepe University Faculty of Pharmacy, Ankara, Turkey

**Keywords:** Anti-aging compounds, astaxanthin, caffeine, curcumin, fucoxanthin, metformin, rapamycin, resveratrol, spermidine, *α*-lipoic acid

## Abstract

Aging is the most prominent risk factor for many diseases, which is considered to be a complicated biological process. The rate of aging depends on the effectiveness of important mechanisms such as the protection of DNA from free radicals, which protects the structural and functional integrity of cells and tissues. In any organism, not all organs may age at the same rate. Slowing down primary aging and reaching maximum lifespan is the most basic necessity. In this process, it may be possible to slow down or stabilise some diseases by using the compounds for both dietary and pharmacological purposes. Natural compounds with antioxidant and anti-inflammatory effects, mostly plant-based nutraceuticals, are preferred in the treatment of age-related chronic diseases and can also be used for other diseases. An increasing number of long-term studies on synthetic and natural compounds aim to elucidate preclinically and clinically the mechanisms underlying being healthy and prolongation of life. To delay age-related diseases and prolong the lifespan, it is necessary to take these compounds with diet or pharmaceuticals, along with detailed toxicological results. In this review, the most promising and utilised compounds will be highlighted and it will be discussed whether they have toxic effects in short/long-term use, although they are thought to be used safely.

## Introduction

Aging can be defined as the accumulation of damage over time, a loss in functional ability, adaptation difficulties to the environment and an increase in illness and mortality. The aging of the global population is posing significant issues for both industrialised and developing countries due to changes in the population pyramid (Refs 1, 2). The percentage of persons over the age of 60 is increasing globally, and it is predicted that by 2050, it would nearly double, rising from 12 per cent in 2015 to 22 per cent (Refs 3–5). Despite the continued predicted increase in the aging population, the geriatric population's life expectancy and survival are improving significantly (Ref. 4).

According to several studies, aging is one of the most important risk factors for chronic diseases including cancer, atherosclerosis, cardiovascular disease, stroke, diabetes, renal failure, chronic lung disease, osteoporosis, rheumatoid arthritis, blindness, dementia and neurological pathologies that cause the greatest global morbidity, mortality and use of medical resources. Additionally, aging increases a person's risk for the geriatric syndrome, a reduction in immunity and slower physical recovery (Refs 5–8).

Due to its significant role as a risk factor for morbidity and age-related diseases, the biology of aging has considerable interest among researchers.

Many hypotheses have been developed to expand our comprehension of the aging process and to help us develop methods for extending life. Based on the level at which the aging mechanism is targeted, the theories of aging are divided into categories including evolutionary, systemic, molecular and cellular theories (Ref. 9).

In aging research, several natural products and dietary supplements continue to be studied extensively for their antiaging properties or are used as research materials to discover molecular mechanisms of biological aging (Refs 10, 11). Using natural resources may be an effective way to delay physiological and pathological aging and, in turn, avoid the development of these disorders, according to recent research (Refs 12–14). Natural compounds that are separated or obtained from plants are distinguished by the synthesis of secondary metabolites and are abundant in nature. Numerous chemical families, primarily flavonoids, phenolic acids and terpenoids, are included in secondary metabolites. Other compounds that are part of the primary metabolites have also demonstrated important anti-aging benefits (Refs 12–14) (Table 1)

**Table 1.** Examples of anti-aging compounds from natural sources^a^ (Refs 15, 16)

Flavonoids	Quercetin, fisetin, kaempferol, acacetin, baicalein, 4,4′-dimethoxychalcone, naringenin, myricetin, dihydromyricetin, epicatechin, nobiletin, rutin, hesperidin, Icariside II, vitexin, theaflavin, taxifolin, icariin, iso-xanthohumol.
Other polyphenols and phenolic acids	***Resveratrol*,** oxy-resveratrol, ***curcumin***, tetra-hydrocurcumin, polydatin, piceatannol, urolithin A, butein, isolappaol A, lappaol C, lappaol F, matairesinol, arctigenin, arctiin, acteoside, ellagic acid, pentagalloyl glucose, tannic acid, chicoric acid, rosmarinic acid, caffeic acid, chlorogenic acid, gallic acid, salicylic acid.
Terpenoids and steroids	***Fucoxanthin***, ***Astaxanthin***, celastrol, oleanolic acid, ginsenoside Rg1, dehydroabietic acid, triptolide, *β*-caryophyllene, catalpol, tomatidine, nolinospiroside F, withanolide A, antcin M, otophylloside B.
Primary metabolites	***α-Lipoic acid*** (a fatty acid derivative), several amino acids.
Others	***Caffeine*** (a methylxanthine derivative), ***metformin*** (a biguanide), ***spermidine*** (an aliphatic polyamine), ***Rapamycin*** (a macrolide lactone), etc.

a Compounds in bold and italic font have been evaluated for their safety and toxicological properties in this review.

Natural products encompass a diverse group of substances made from a variety of sources, including marine organisms, bacteria, fungi and plants. Before the market, the safety of natural products is sometimes under- or unstudied, which raises the possibility of unpleasant responses with the natural products alone as well as with accompanying medications and other natural products. For anticipating and reducing natural product-drug interactions and adverse events, standardised procedures and in vitro testing have not been developed and specialised clinical research may be prohibitively expensive. In addition, some natural products contain ingredients or additives that are unidentified and uncharacterised, which poses a serious risk to users (Ref. 17).

These compounds and their active components work include tissue formation, telomere activation, anti-senescence action, DNA repair and targeted antioxidant activity. In this review, the evidence supporting the anti-aging properties of some of the most promising natural substances in this area will be discussed and evaluated by summarising pharmacological and toxicological properties.

## Molecular mechanisms of aging

Significant influences on aging can be found in genetics, the environment and intrinsic variables. Common age-related functional loss and an increase in multimorbidity with age are caused by damage accumulation (Ref. 18). Human aging is a progressive time-related process. It varies among individuals and, biologically, relates to a loss of homoeostasis, an increase in the organism's sensitivity and susceptibility to disease and death, as well as the gradual aging-related deterioration of cells, tissues and organs. Senescence refers to these features of aging and is what causes a person's health to deteriorate. Additionally, they contribute to age-related disease including coronary heart disease and physiological changes like diminished renal function in aging (Refs 19, 20).

As a result of a genetic programme, the loss of homoeostasis is eventually caused by a combination of factors. Changes in mitochondria, the accumulation of lipofuscins that cause cellular aging, changes in the cellular structure or macromolecular aggregation at the molecular level, telomere loss and shortening at the chromosomal level and amyloid deposition are all monitored parts of the aging process. As a consequence of genetic changes, the phenotype, functionality and behaviour of an organism can be affected (Ref. 14).

Researchers claim that aging is a physiological deterioration caused by damage to essential molecules in aging populations (Refs 21, 22). The aging process differs greatly from aging in a single organism, tissue or cell. While studying the various genes involved in senescence, which can be categorised into three groups: genes regulating somatic maintenance and repair, genes promoting early survival and genes causing late deleterious mutations, other academics have concentrated on understanding the evolutionary foundation of senescence in the aging mechanism. Due to their increased development and repeatability, these critical genes have been found to affect a species' evolution and survival, and they may also contribute to shortened lifespans. Cellular senescence may have a pleiotropic effect that prevents cancer while also speeding up the aging process of the body (Refs 23, 24). It is known that aging is associated with hallmarks of aging: genomic instability, telomere attrition, epigenetic alterations, loss of proteostasis, deregulated nutrient-sensing, mitochondrial dysfunction, cellular senescence, stem cell exhaustion and altered intercellular communication (Ref. 25).

The accumulation of genetic damage, which may interfere with cell homoeostasis and lead to genomic instability, is one widely accepted factor in aging. Copy number mutations, somatic mutations and chromosomal aneuploidy all contribute to escalating DNA damage. Age-related abnormalities in the DNA repair process influence the expression of vital genes and transcriptional pathways, which in turn causes cell malfunction (Refs 8, 26). Numerous aging models have implications for the various processes that can cause transcriptional alterations related to aging. Since mutations in genes like daf-2 or daf-16, which encode the IGF-1 receptor and FOXO, respectively, can lengthen worm lifetime, aging can be controlled (*Caenorhabditis elegans*). Additionally, there are clear links between lifespan and genetic loci such as APOE, FOXO3, 5q33.3 and ACE (Ref. 27). Premature aging disorders have been linked to impaired DNA repair capacity, according to different preclinical investigations (Refs 8, 26). DNA helicases are crucial components of the response to DNA damage. Since several human RecQ helicases are deficient in conditions linked to cancer and premature aging, the RecQ family of DNA helicases is of great interest (Ref. 28).

Telomere length is linked to age-related diseases and DNA damage, and telomere shortening can affect somatic stem cell activity. Human somatic cells have low levels of telomerase expression, which causes telomere shortening and replicative senescence. The typical length of the terminal restriction fragment of chromosomes diminishes with age in fibroblasts and peripheral blood cells (Refs 29, 30). These results demonstrate that the most important factor of cellular aging is telomere length, but not telomerase activity. Furthermore, rather than the average length of telomeres, the shortest telomere affects cell viability and chromosome stability. In this regard, numerous investigations have shown that telomere lengthens with age in both humans and animals (Refs 29, 31). Telomere length affects mortality risk and replicative senescence, which stops cancer cells from proliferating forever. Telomere shortening continues to be a characteristic and a counting mechanism of senescent cells (Refs 1, 14).

Another potential factor in aging is the discovery that the bulk of age-related changes is brought on by free radical atoms or molecules that have an unpaired and reactive electron. These oxygen-derived species are secondary messengers in signalling pathways involved in the regulation of various mechanisms, including changes in gene expression, cell replication, differentiation and apoptotic cell death (Refs 32, 33). They can also react with macromolecules to produce free radicals from the molecules that were attacked. Maximal lifespan is impacted by the production of these free radicals in human organs such as the heart, kidney and liver. In this situation, nutritional antioxidants have been shown to lower the risk of cancer, heart disease and dementia. For instance, higher dietary intake of vitamin C, carotenoids and *α*-tocopherol was associated with reduced risk of cardiovascular disease, cancer and all-cause mortality. Reactive oxygen species (ROS) itself, which are one of the developmental-genetic features of aging, contribute to the somatic accumulation of mutations in mitochondrial DNA. These mutations cause aging and cell death, as well as a gradual reduction of bioenergetic capacity (Refs 14, 34, 35).

Epigenetic alterations play also an important role in the aging process. DNA methylation, histone changes and chromatin remodelling are chromatin modifications that are connected to cell aging. Through modifications to the chromatin structure and DNA accessibility to transcription factors, epigenetic processes control gene expression. On the one hand, the epigenetic alterations linked to senescence function as compensatory mechanisms that let cells resist growing stress and avoid irreparable DNA damage. On the other hand, the modifications result in side processes that hurt cells and the body as a whole, speeding up aging and the emergence of age-related disorders (Refs 1, 8).

A genetically designed continuum of growth and maturation is thought to be the primary driver of the aging process. The maximum lifespan is very species-specific, as humans have a maximum lifespan 30 times longer than mice. Natural resources present a great potential for searching for longevity features. In this regard, it has been shown that the complete lifespan potential is determined by a set of particular genes that are present in a wide variety of species. These genes cause the synthesis of substances that are involved in the regulation of the species' life through a variety of methods, including the modulation of stress and resistance, the increase in metabolic capacity and the silencing of genes that promote aging (Refs 14, 36).

The disturbance of protein homoeostasis is also linked to the onset of aging and most aging-related illnesses. Almost all tissues of older organisms, particularly those with a low rate of proliferation, include modified and misfolded proteins, as well as protein, aggregates Aging symptoms include protein aggregation, post-translational modification and altered protein turnover. Proteostasis is distorted by nonenzymatic posttranslational changes that accumulate with aging in all cell compartments. The process of nonenzymatic glycosylation (glycation), which results in the production of advanced glycation end products, hazardous intermediate products, and crosslinks between protein molecules, is best understood in the context of aging (Refs 1, 14).

Recent investigations have shown that bioactive compounds from different medicinal and nutritional species exhibit remarkable anti-aging effects. According to a comprehensive review by Ding *et al*., 185 natural compounds and 55 complex/extracts among the approximate 300 longevity compounds were revealed. The selected popular anti-aging natural and pharmaceutical products have been evaluated below regarding their safety and toxicological properties. These molecules showed antiaging properties in at least two aging models (Ref. 15). Model organisms such as the nematode *Caenorhabditis elegans*, the fruit fly *Drosophila melanogaster*, the mouse *Mus musculus*, and the yeast *Saccharomyces cerevisiae* were mostly used for the explanation of the longevity properties of candidates (Ref. 11). Table 2 shows the average percentage of the mean life-prolonging effects of the compounds of interest in different models.

**Table 2.** The longevity effects of the selected compounds

Compound	Experimental model	Treatment dose	Results	Ref.
Caffeine	*S. cerevisiae* *C. elegans*	0.2–0.4 mM 0.1% 10 *μ*M	Extension in median survival of wild type by 0.86 52% increase in maximum lifespan 36.7% extension in mean lifespan	(Ref. 37) (Ref. 38) (Ref. 39)
Metformin	Mice *C. elegans*	0.1% w/w in diet 50 mM	5.83% extension of mean lifespan 40% increase in median survival	(Ref. 40) (Ref. 41)
Fucoxanthin	*D. melanogaster* *C. elegans*	0.3–1.0 *μ*M 5 *μ*M	33–49% increase in the median lifespan of females; 33% increase in those of males. 14.0% increase in the mean lifespan and 14.0% increase in maximum lifespan.	(Ref. 42)
Spermidine	*D. melanogaster* *C. elegans:*	0.01 mM, 0.1 mM, 1 mM 0.2 mM	Up to 30.0% increase in mean lifespan Up to 15.0% increase in mean lifespan	(Ref. 43)
Rapamycin	Mice *D. melanogaster*	2.24 mg per kg body weight per day 42 ppm 200 *μ*M	14.0% increase for females and 9% for males 23% increase in median lifespan in males and 26% in females 13.0% increase in median lifespan	(Ref. 44) (Ref. 45) (Ref. 46)
*α*-Lipoic acid	*D. melanogaster C. elegans*	0.005% 24 *μ*M	12.0% increase in average survival time of females, 4% for males 24.0% increase in mean lifespan and 14% increase in maximum lifespan	(Ref. 47)
Resveratrol	*D. melanogaster C. elegans*	200 and 400 *μ*M 200 *μ*M 100 *μ*g/ml	14.6% extension in females fed the low sugar-high protein; 10.0% extension in those fed the high-fat diet, respectively (Canton S strain). 11.4% and 12.8 extension in females and males fed the high-fat diet, respectively (sod1RNAi strains). 11.0% and 11.5% increase in mean and maximum lifespan, respectively.	(Ref. 48) (Ref. 49)
Astaxanthin	*D-galactose induced brain aging rats* *C. elegans*	0.02% of daily diet three times weekly Continuous treatment with 0.1 to 1 mM	Antioxidation and restored BDNF expression 16–30% extension in mean of lifespans in the wild-type N2 and long-lived mutant *age-1*.	(Ref. 50) (Ref. 51)
Curcumin	*D. melanogaster C. elegans*	0.5 and 1.0 mg/g of diet 100 *μ*M	6.2% and 25.8% increase in mean lifespan for females 12.62% extension in mean survival and an increase of 27.77% in maximal survival.	(Ref. 52) (Ref. 53)

## Safety and toxicological properties of selected longevity compounds

### Caffeine

**Figure 1.**
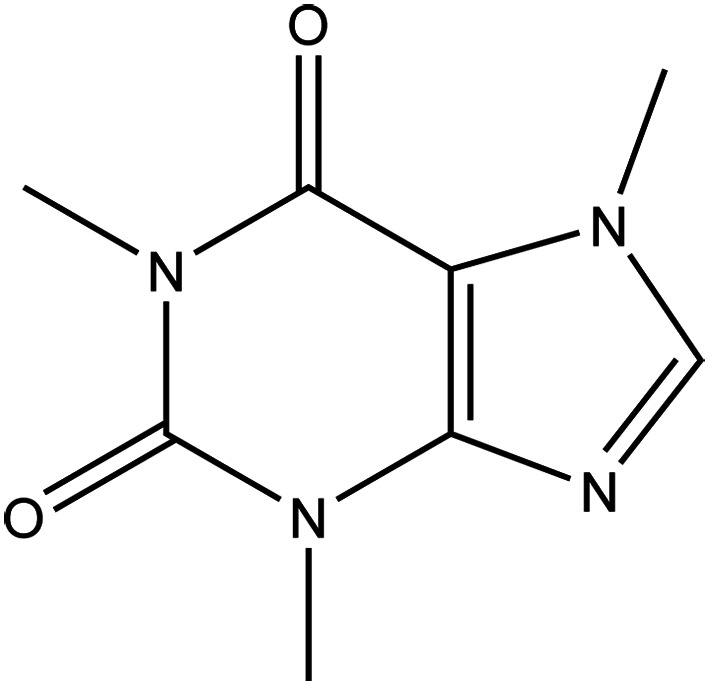
The chemical structure of caffeine.

Caffeine (Fig. 1), a methylxanthine derivative (13,7-trimethylxanthine) is a secondary metabolite classified as a purine alkaloid and synthesised majorly in various plants such as *Coffea arabica* L. (Arabica coffee), *Camellia sinensis* (L.) Kuntze (tea), *Paullinia cupana* Kunth (Guarana) and *Cola* sp. (Ref. 54). Psychomotor stimulant properties of caffeine are primarily associated with its reducing properties on adenosine transmission via the blockade of adenosine A_2A_ in the brain (Ref. 55). Other mechanisms of action proposed for caffeine, inhibition of phosphodiesterase and mobilisation of intracellular calcium, require high concentrations of caffeine than likely to be taken in daily consumption (Ref. 56).

Today, caffeine is used as an adjuvant in some analgesics or as a major constituent in some energy beverages (Refs 57, 58). The FDA and EFSA reported that habitual consumption of 400 mg of caffeine (approximately 4 or 5 cups of coffee and about 5.7 mg/kg body weight (BW) per day for a 70-kg adult) throughout the day can be considered safe except for pregnant women, however, it should be considered that may differ according to individual sensitivity, lactating, medication use, etc. (Refs 59, 60). Single doses of caffeine up to 200 mg (about 3 mg/kg BW for a 70-kg adult) in healthy adults (or when consumed less than two hours before an intense physical exercise) do not give rise to health concerns. Habitual consumption and a single dose of caffeine for adults may also consider safe for children and adolescents because of similar caffeine clearance (Ref. 60).

It has been suggested that age-related neurodegenerative diseases are reduced by chronic, moderate caffeine consumption as well as habitual consumption was correlated with reduced mortality and positively affected healthspan features. In addition, its lifespan extending effect on the worm model was reported (Ref. 39). However, National Health and Nutrition Examination Survey on 5826 adults from two genders, investigating the relationships between caffeine and coffee intakes, and telomere length, presented that caffeine consumption caused a tendency to shorten telomeres in US adults. However, telomere lengths were positively affected by coffee consumption (Ref. 61).

After intake of therapeutic amounts of caffeine, hypertension can be noted, however, hypotension is frequently noted in the case of overdoses. Acute toxicologic symptoms occur with 15 mg/l and higher plasma concentrations of caffeine, while 50 mg/l is considered toxic. Generally reaching ≥ 80 mg/l of plasma concentrations, symptoms indicating the lethal phase can be observed. The clinical symptoms of caffeine intoxication from mild to lethal level have included nausea, vomiting, agitation, anxiety, seizures, delusions, hypokalemia, rhabdomyolysis, hypertension, hypotension, tachycardia, bradycardia, myocardial infarction, cardiac arrest, respiratory and renal failure and death. Daily intakes of 1 to 1.5 g of caffeine can result in chronic toxicity of which symptoms are similar to chronic anxiety (Ref. 62). Besides 2 g of caffeine ingestion requiring hospitalisation, ≥5 g of caffeine ingestion is estimated to be an overdose in adults (Ref. 63).

Maximum plasma concentration of caffeine is reached in 15–120 min after ingestion. Caffeine is mainly metabolised to its pharmacologically active but less toxic metabolite paraxanthine (1,7-dimethylxanthine) by cytochrome P450 1A2 (CYP1A2) in the liver. Caffeine is also metabolised to 1-demethylated product theobromine and 7-demethylated product theophylline, which are also pharmacologically active, by CYP1A2 (Ref. 62). Thus, possible metabolic interaction with CYP1A2 may change the metabolism of caffeine. For example, the potent inhibitory effect of the selective serotonin reuptake inhibitor fluvoxamine on CYP1A2 reduces the clearance of caffeine during concomitant intake which may lead to caffeine intoxication (Refs 64, 65). Yet another example, antibacterial quinolone derivatives, enoxacin, pipemidic acid, ciprofloxacin and norfloxacin, competitive and dose-dependent inhibitors of CYP1A2 enzyme, have been reported for their AUC-enhancing and caffeine clearance-reducing effects (Ref. 66). 5-methoxypsoralen (bergapten) and 8-methoxypsoralen (methoxsalen), which are naturally occurring linear furanocoumarins and used to treat psoriasis, inhibit the metabolism of caffeine by CYP1A2 mediated-inhibition (Ref. 66).

### Metformin

**Figure 2.**
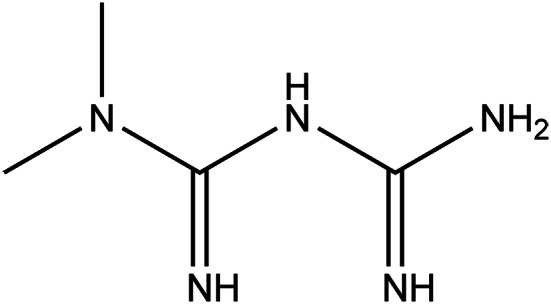
The chemical structure of metformin.

Metformin (MET, Fig. 2) is a biguanide used as a first-line treatment for type-2 diabetes mellitus (Ref. 67). The discovery of MET (dimethylbiguanide) is linked to a plant *Galega officinalis* L., which was traditionally used in Europe to treat the symptoms of diabetes and was later found to be rich in guanidine with blood sugar-lowering effects. The synthesised guanidine derivative MET was introduced as an antidiabetic agent in the 1990s in the USA (Ref. 68). It is a potent antihyperglycemic agent, which counters insulin resistance, reduces hepatic gluconeogenesis and increases glucose uptake, without causing weight gain and overt hypoglycemia (Ref. 69). The drug also has beneficial features for diabetes-related polycystic ovary disease, fatty liver disease and cardiovascular complications, alongside being suggested as an adjuvant treatment for cancer or gestational diabetes in the pre-diabetic population (Ref. 70).

The enhancing effects of MET on insulin sensitivity are assigned to its stimulation of the tyrosine kinase activity of the *β* subunit of the insulin receptor (Ref. 69). At the molecular level, MET blocks the mitochondrial respiratory chain, which leads to the activation of AMPK resulting in suppression the production of gluconeogenic enzymes (Ref. 71). MET's potential as an anti-aging candidate was shown in several studies. In the context of telomere length, telomere shortening was reduced by metformin in the mild age-related diabetes (MARD) group. The telomere length of MARD group was significantly shorter than the NON-MARD group, however, was disappeared after MET (Ref. 72).

MET circulates in the plasma unbound and is excreted unchanged in the urine. The bioavailability of the MET formulations is about 50–60% (Ref. 73). Pharmacokinetic distribution of MET is mediated by organic cation transporters (OCT1, OCT2 and OCT3), multidrug and toxin extruders (MATE1 and MATE2-K) and plasma membrane monoamine transporter (PMAT). Oral absorption, hepatic and renal uptake are mediated by OCTs. Renal excretion is mediated by OTC2 and MATEs (Refs 67, 74, 75). As MET is not metabolised and excreted unchanged, drug-drug interaction is not expected for MET. However, inhibition of MET transporters, which could be resulting in increased plasma concentration, is associated with drug interactions. (Refs 74, 75). Increased plasma concentration and AUC of MET were observed when co-administration with the proton pump inhibitors, lansoprazole, pantoprazole and rabeprazole, as well as a decrease in renal excretion by 13%, and prolonged elimination half-life of MET from 3.9 to 4.5 h accompanied by lansoprazole. Thus, MET and lansoprazole together were suggested as a risk for those with lactic acidosis (Ref. 76).

The most frequent side effects of MET are gastrointestinal defects. MET-associated lactic acidosis, suggests being contraindicated in those who have substantial renal dysfunction, and anaemia due to vitamin B12 malabsorption and deficiency has been rarely reported (Ref. 76). The overdose administration of this drug results specifically in metabolic acidosis and hyperlactatemia, which are indicated to be the markers of metformin toxicity. Metformin attracts attention with a secondary effect on weight loss, for this reason causing long-term drug abuse. A report serves metformin intoxication symptoms such as cardiogenic shock and hypotension, cardiac dysrhythmia and ultimately cardiac arrest (Ref. 77). Another study on patients with type 2 diabetes concludes that vitamin B12 deficiency is a potential side effect in long-term and high-dose metformin therapy (Ref. 78). Studies categorised metformin overdose toxicity into two groups: MALA (metformin-associated lactic acidosis) and MILA (metformin-induced lactic acidosis). Clinical reports also remark on hypoglycemia accompanying metabolic acidosis but the toxic dose has not been determined yet (Ref. 79).

### Fucoxanthin

**Figure 3.**
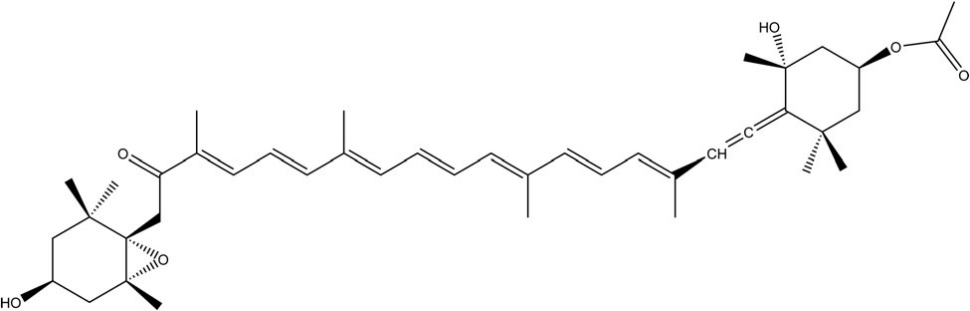
The chemical structure of fucoxanthin.

Fucoxanthin (Fig. 3) is a naturally occurring marine carotenoid, which was found in algae such as *Undaria pinnatifida*, *Laminaria japonica*, *Phaeodactylum tricornutum* and *Cylindrotheca closterium*, and has a unique structure. The molecular structure, which was composed of a conjugated carbonyl group with an unusual allenic bond, a 5,6-monoepoxide and some oxygenic functional groups, is unstable and easily degradable by oxygen, heat, light, etc (Ref. 80). *Trans*-fucoxanthin, which is responsible for its biological activities, is shown to produce its two minor *cis*-isomers according to storage conditions (Refs 81, 82).

Fucoxanthin has been shown to have potential health benefits for lifestyle-related diseases by the evidence of hepatoprotective, anti-inflammatory, antioxidant, antidiabetic, antitumoural and anti-obesity activities (Ref. 80) along with anti-aging properties. Especially with its enhanced antioxidant capacity, fucoxanthin inhibits the ROS which increases in some diseases like ischaemic stroke and acute lung injury (Ref. 83).

Fucoxanthin is hydrolysed into fucoxanthinol through deacetylation by digestive enzymes, lipase and cholesterol esterase in the gastrointestinal tract before absorption, and fucoxanthinol is later converted into amarouciaxanthin A by short-chain dehydrogenase/reductase in the liver (Ref. 84). The pharmacokinetics of fucoxanthin depends on the species (Refs 81, 82). Fucoxanthinol did not show significant adverse effects at 200 mg/kg BW in C.B-17/Icr-SCID mice for 28 days (Ref. 85). Several studies on this bioactive compound containing extracts have been performed to determine the short- and long-term toxicity and repeated oral dose toxicity as well. No significant toxicity and abnormalities have been observed in rats and mice after 14, 30 and 90 days of administration (Ref. 86).

Orally administrated fucoxanthin at the doses of 500, 1000 and 2000 mg/kg did not cause any mutagenicity in mice. In addition, fucoxanthinol, the major metabolite of fucoxanthin after oral administration, was tested with the *in vitro* Ames test, in which no mutagenicity was detected under the experimental condition (Ref. 87)

Fucoxanthin can be accepted as a safe product. In a 14-day single-dose study (1000 and 2000 mg/kg) and a 30-day repeat-dose study (500 and 1000 mg/kg) in ICR mice, no abnormalities and mortalities were shown. However, increased total cholesterol concentrations were observed by plasma biochemical analyses in all fucoxanthin-treated groups (Ref. 88). In a double-blind placebo-controlled study on Japanese obese subjects, 1 or 3 mg daily fucoxanthin capsule for 4 weeks exhibited no side effects (Ref. 89). The Panel on Dietetic Products, Nutrition and Allergies of the European Commission gave an opinion about the consumption of the thallus part of *Undaria pinnatifida*, which has to carry an amount equivalent to 15 mg fucoxanthin per day. However, this food source has not enough evidence on the maintenance or achievement of normal body weight as claimed by the manufacturer (Ref. 90). Although there is a lack of evidence on the safety of fucoxanthin consumption, the Food and Drug Administration announced fucoxanthin extracted from the alga, *Phaeodactylum tricornutum*, as a new dietary ingredient that can be consumed at a level of 3 mg/day with no time limit or 5 mg/day fucoxanthin for up to 90 days (Ref. 91). It is obvious that to present a safety profile for the supplementation levels of fucoxanthin in humans, reported clinical trials remain limited.

### Spermidine

**Figure 4.**
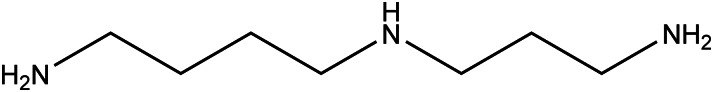
The chemical structure of spermidine.

Spermidine (Fig. 4) is a natural aliphatic polyamine and is found in all living cells of plants, animals and microorganisms, as well as can also be found in plant- and animal-originated foods (Ref. 92). For several organisms, the proportion of polyamines like spermidine is due to either administration via dietary sources or synthesised by the gut microbiota (Ref. 93). Spermidine is found high in foods such as wheat germ, fermented soybeans (Natto), mushrooms, nuts and some fruits and vegetables. It is absorbed mainly in the duodenum and proximal jejunum parts of the small intestine (Ref. 94). The nutritional intake of foods that contain arginine (precursor of polyamines) helps polyamine-producing bacteria produce spermin and spermidine in the microbiota of mammals (Ref. 95). The studies suggest that a polyamine rich diet including spermidine helps humans reach older ages healthy, recover especially after surgery and wound healing because of its antioxidant properties (Refs 92, 93). It has antitumour, cardiovascular protective, neuromodulator, anti-obesity and anti-inflammatory features, and is an autophagy-inducing agent, as well (Refs 92, 94, 95). Besides, spermidine treatment induced a reduced percentage of the nuclei with short telomeres (Ref. 96).

An *in vivo* study, 0.5, 5 or 50 g/kg mice BW daily intake over 28 days, and a 3-month randomised, placebo-controlled, double-blind Phase II (at the dose of 1.2 mg/day) study showed that spermidine rich wheat extract to be safe and well tolerable by mice and older human (Ref. 97). Til *et al*. determined the acute oral toxicity of spermidine at 600 mg/kg BW in Wistar rats. A significant increase in plasma activities of alkaline phosphatase, alanine aminotransferase and aspartate aminotransferase at the dose of 10.000 ppm and decrease in plasma protein level in female rats; and a significant decrease in food intake and body weight along with plasma calcium and potassium levels in male rats were observed (Ref. 98). The homoeostasis of polyamines in the mammalian tissue is regulated by several functions including catabolic pathways. According to the studies, the catabolic reactions may result in some toxic products such as acrolein and other reactive aldehyde species, which are being catalysed by spermidine/spermine N(1)-acetyltransferase and serum polyamine oxidase (Ref. 99). Although spermidine is an abundant polyamine in mammalian cells and has lower toxicity than other polyamines like spermine, experimental studies show that these byproducts may be harmful to patients with cancer and chronic renal failure (Ref. 95). However, the toxic dose for humans as supplementary spermidine has not been determined yet (Refs 100, 101).

Successive development of spermidine-related products as natural dietary supplements (SpermidineLIFE^®^ Immunity+, SpermidineLIFE^®^ Original 365+, SpermidineLIFE^®^ Memory+) and launch on the market in 2019 suggests the safety of spermidine in food supplements (Refs 102, 103)

### Rapamycin

**Figure 5.**
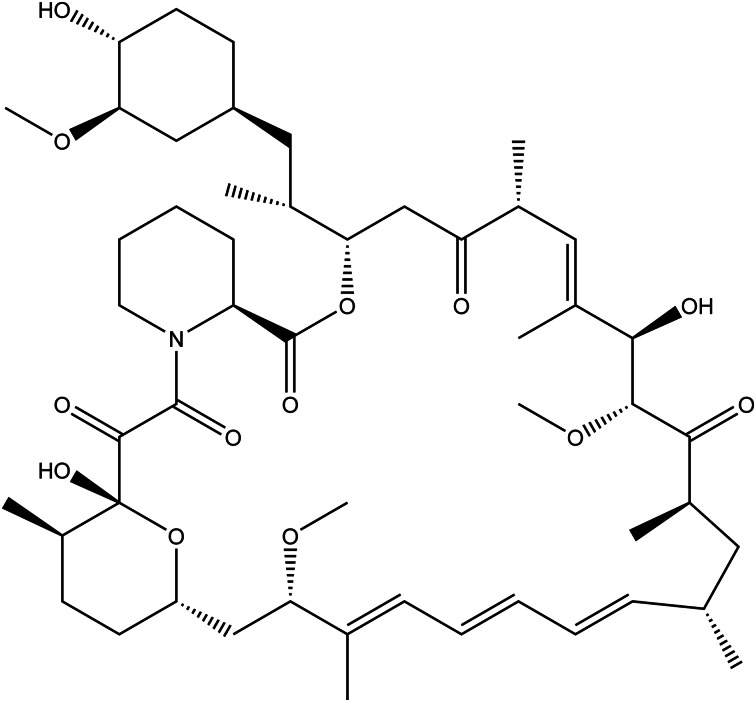
The chemical structure of rapamycin.

Rapamycin (RAPA, Fig. 5), also known as FK506 or sirolimus, a lipophilic macrolide lactone was initially isolated from *Streptomyces hygroscopicus* from a soil sample found on Easter Island (Rapa Nui). The antifungal activity of RAPA, particularly against *Candida albicans*, was first demonstrated, and immunosuppressive and anticancer properties were later proven (Refs 104, 105). FDA approved rapamycin according to results of the 2 double-blind clinical trials at the fixed doses of 2 and 5 mg/day with co-administration of cyclosporin and corticosteroid prednisone, for use in kidney transplant (Refs 59, 106). It is now a prescription drug named Rapamune^®^ for preventing tissue rejection after kidney transplantation for people ≥13 years old, but not recommended for liver and lung transplant patients (Ref. 107).

The immunosuppressive features have been shown to procure at the plasma concentration of 5 to 20 *μ*g/l (Ref. 106), and 5–15 *μ*g/l is recommended for patients at standard risk of rejection (Ref. 108). Rapa is shown to be at the highest concentration in the red blood cells at the ratio of 94.5%, and the remaining drug is distributed among the plasma (3.1%), lymphocytes (1.01%) and granulocytes (1.0%) (Ref. 106).

Rapamycin acts as an immunosuppressant through the inhibition of the mammalian target of rapamycin (mTOR) by binding to its intracellular receptor FK506-binding protein 12 (FKBP12). mTOR is inhibited via the interaction of the FKBP12-rapamycin complex directly with the relevant domain of mTOR (Ref. 109). mTOR inhibition is also regarded to be important for preventing aging and age-related disease. However, a recent finding demonstrated that mTOR is upregulated acting as a survival pathway in mice with short telomeres, thus the inhibition of this pathway was found to be harmful to telomere-deficient mice (Ref. 110).

RAPA is metabolised by the cytochrome CYP3A4/P-glycoprotein system, which is mainly localised in the liver and intestine that may be responsible for the first pass effect resulting in low drug bioavailability (~14%) in oral administration. (Refs 108, 111). 4′-*O*-demethyl, 7-*O*-demethyl, several hydroxy, dihydroxy, hydroxydemethyl and didemethyl sirolimus, which do not serve an important contribution significantly to the activity of the parent drug, were characterised as metabolites of RAPA (Ref. 112).

Strong CYP3A4/P-glycoprotein inhibitors clinically increase the RAPA concentration. Antifungal azoles; corticosteroids; calcineurin inhibitor cyclosporine; non-dihydropyridine calcium channel blocker diltiazem was shown to inhibit the CYP3A4 in the pharmacokinetic studies, leading the increased RAPA levels in blood (Ref. 113). Cyclosporine is also a P-glycoprotein inhibitor, and in the case of concurrent treatment, RAPA is recommended to be taken 4 hours after cyclosporine administration. In addition, ketoconazole, voriconazole, itraconazole, erythromycin, telithromycin and clarithromycin are strong inhibitors of CYP3A4/P-glycoprotein as well as grapefruit Juice inhibits the CYP3A4-mediated metabolism of RAPA (Ref. 107).

Some antiaging studies about rapamycin on mice revealed unexpected side effects such as insulin resistance, testicular degeneration and ophthalmic problems (Refs 114–116). Clinical studies confirm the *in vitro* and *in vivo* findings about the adverse effects of rapamycin treatment on peripheral insulin insensitivity, by showing deleterious effects on pancreatic *β*-cells with the inhibition of mTORC1 and mTORC2 complexes (Ref. 117). A cohort study on male C57BL/6J mice testing rapamycin on healthy aging resulted in some side effects on the kidney and reproductive tract compared to the control group. In addition to nephrotoxicity and gonadotoxicity, impaired glucose tolerance and hypercholesterolaemia were observed on rapamycin-treated animals after treatment with 14 mg/kg encapsulated rapamycin in food for 1 year (Ref. 115). Ross *et al*. studied long-term rapamycin exposure on marmoset monkeys (*Callithrix jacchus*) to clarify the underlying mechanism of hyperglycaemia, also seen in rodent studies. In contrast with the other animal models, hyperglycaemia was not seen in healthy marmosets. It was concluded that one of the theories was the increase in hepatic gluconeogenesis (Ref. 118). In a study on 24 middle-aged healthy dogs, non-immunosuppressive doses (0.05 or 0.1 mg/kg orally three times per week) of rapamycin for 10 weeks did not cause significant clinical side effects or abnormal haematological changes. However, corpuscular volume (the volume of the red blood cells) was significantly decreased in RAPA-treated groups (Ref. 119).

Serious allergic reactions such as swelling on the face, eyes or mouth, trouble in breathing, chest pain or tightness, rash or peeling of the skin, feeling dizzy or faint, chest pain or tightness; edema; poor wound healing, increased levels of cholesterol and triglycerides; increased protein in the urine; increased risk for viral infections, lung and breathing problems are indicated as the possible serious side effects of the drug Rapamune^®^ (Ref. 120). The nephrotoxicity in a clinical study appeared on not healthy people but patients with renal health problems. More detailed mechanism studies needed to be discussed to evaluate the side effects of rapamycin in the treatment dose in humans (Ref. 121).

### *α*-Lipoic acid

**Figure 6.**
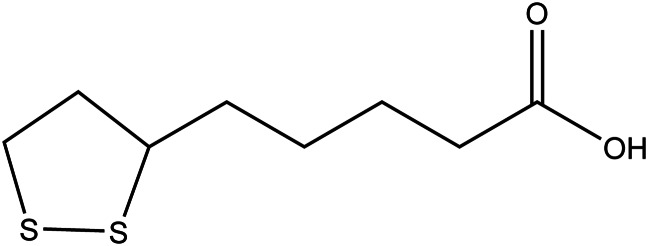
The chemical structure of *α*-lipoic acid.

*α*-Lipoic acid (*α*-LA, Fig. 6), also called thioctic acid (CAS RN 1077-28-7), is a caprylic acid-derived fatty acid found in several dietary sources such as red meats, heart, kidney, liver, wheat germ and to a lesser degree, fruits and vegetables. The compound has a dithiol functional group, which reacts with free radicals (Ref. 122). The studies reveal that it provides detoxification and promotes some biochemical reactions, especially the renewal of damaged detoxification enzymes that helps to treat chronic diseases associated with oxidative stress. Both *in vitro* and *in vivo* studies have shown *α*-lipoic acid demonstrating very high radical scavenging activity, the main reason for which has been added as an ingredient in several dietary supplements such as multivitamins also used by intravenous injection. Especially its reduced metabolite dihydrolipoic acid (DHLA) showed quite high radical scavenging activity (Ref. 123). PGC-1*α* (peroxisome proliferator-activated receptor *γ* coactivator-1*α*) is a receptor, plays a role in protecting from age-related chronic diseases. Xiong et.al. 2005 indicates that *α*-LA, as a cofactor, upregulated PGC-1*α*-dependent-TERT (telomerase reverse transcriptase), therefore modulating age-dependent arteriopathy which helps reducing vascular aging (Ref. 124). Studies also indicate that it is not possible to keep enough levels of *α*-LA only from natural sources to see its anti-aging effect, so to have significant antioxidant activity it is needed to take *α*-LA as a supplement in higher doses. Therefore, the need of assessing its daily dose for humans occurred in the light of acute and subchronic toxicity, and potential mutagenic/genotoxic activity. Studies currently have found no adverse effects in the acute toxicity studies when given 175–550 mg/kg BW *α*-LA by oral gavage for 14-days to female CD Sprague–Dawley rats. At 2000 mg/kg BW, some rats ‘were reported to show signs of reduced well-being, including sedation, apathy, piloerection, hunched posture and/or eye closure. There was no effect of treatment observable on body weight gain or gross pathological examination. As a result of the study, the acute oral LD_50_ of *α*-LA was concluded to exceed 2000 mg/kg (BW), the highest dose tested in the study which indicates a very low order of acute toxicity. A 4-week sub-chronic toxicity study on both male and female Wistar rats was performed with the determined doses from low (31.6 mg/kg BW/day) to high (121 mg/kg BW/day). The no-observed-adverse-effect level (NOAEL) is considered to be 61.9 mg/kg BW/day. The results of these studies support the safety of *α*-LA (Cremer *et al*. 2006). The experimental safety dosage values were found 400 to 500 mg/kg for dogs, 30 mg/kg for cats and 500 mg/kg for mice (Ref. 123).

Shay *et al*. 2019 compiled a series of clinical trials [ALADIN (I, II and III), SYDNEY (I and II) and ORPIL] using *α*-LA which also assessed adverse health effects in the participants. Oral doses of 1800 mg LA (600 mg t.i.d.) for 6 months did not elicit significant adverse effects compared to placebo (Ref. 123). However, there are a series of studies that determined increased plasma lipid hydroperoxide levels and oxidative protein damage with the intraperitoneal administration of racemic LA (100 mg/kg BW/day for 2 weeks) in aged rats (Refs 125, 126). When the equivalent dose is calculated (5 to 10 g per day in humans) it can be seen as too high for human administration. In a systematic review and meta-analysis of randomised placebo-controlled clinical studies, Fogacci *et al*. 2020 discussed *α*-LA ‘safe’ as a supplement to improve health outcomes in overall healthy individuals and patients affected by other diseases (Ref. 127).

Although its therapeutic potential, drug/herbal interactions and adverse reactions have not been well established in clinical trials with populations at higher risk for diseases of aging (Ref. 128). *α*-LA has a low risk in prolonged use, however, according to two published case reports, scientists have drawn attention to serious *α*-LA acute intoxication by suicidal intention with doses of 18 and 6 g respectively (Refs 129, 130). The authorities concluded that *α*-LA can be considered safe as supplementation without any side effects with a range of daily doses ranging from 200 to 2400 mg/day. However, monitoring is advised when taking high doses of *α*-LA frequently (Ref. 123). Researchers conclude that for the safety and optimal dose of *α*-LA more studies are needed.

Since there is not any established contraindication of *α*-LA in pregnant women or children, it has been advised to consult with their healthcare providers before taking *α*-LA, considering the lack of evidence (Refs 122, 131). Even though *α*-LA intoxication is extremely rare, close monitoring is necessary for children who are around diabetic patients, as most of the reported *α*-LA intoxication cases belong to children ingesting *α*-LA by accident (Ref. 122).

Drug interactions: Patients with type 2 diabetes commonly intend to use dietary/herbal supplements to help decrease blood sugar. In the case of regarding a possible risk of herbal-drug interaction, patients who use metformin should avoid using *α*-LA as a supplement because of the synergistic effect (Ref. 132).

### Resveratrol

**Figure 7.**
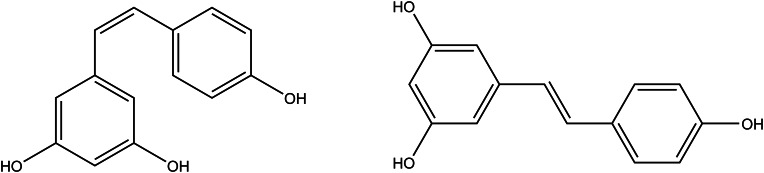
The chemical structures of *cis*-resveratrol and *trans*-resveratrol.

Resveratrol (35,4′- trans-hydroxystilbene, Fig. 7) is a plant-specific polyphenolic compound produced by numerous plant species generally synthesised in terms of some stress conditions in fruits (especially grapes and several berries), nuts and table wines. The fruits of *Vitis vinifera* (grape) are dedicated as the most important dietary source of resveratrol (Ref. 133). It also belongs to the phytoalexin group because it has occurred under some stress conditions such as microbial infections, intense radiation and heavy metals (Ref. 134). The importance of resveratrol as a supplement for human health comes from several study results showing antioxidant, anticarcinogenic and antitumour activity (Ref. 135). The radical scavenging mechanism of resveratrol can be explained as acting as a pro-oxidant first, stimulating the free radical generation and then eliminating the ROS. This phenomenon is called ‘hormesis’ (Ref. 136). Besides its high antioxidant capacity studies showed that resveratrol activates proteins such as Sir2/SIRT1 and AMPK, which modulate lifespan in organisms (Ref. 137). Resveratrol could be a potential therapeutic supplement in preventing long-term cardiovascular morbidity and mortality (Ref. 138). Studies claim that resveratrol also significantly increased telomerase activity *in vitro*. Resveratrol has been shown to delay endothelial progenitor cells senescence by increasing the number of the cells, which may be dependent on telomerase activation (Ref. 139). Resveratrol was proven to prolong the lifetime with a range of 10 to 72% in several study models (Ref. 11). Resveratrol has *cis*- and *trans*- isomeric forms, of which numerous studies have focused on the biological activities and safety studies of *trans*-form (Refs 135, 140, 141). *trans*-Resveratrol has been found more stable with high bioactive effects (Ref. 135). According to the studies, resveratrol increases longevity and provides recovery for age-related diseases via scavenging free radicals (Refs 15, 142). Together with rapamycin and curcumin, resveratrol is in the preclinical test phase of the ‘Interventions Testing Program of the National Institute on Aging’ to be a candidate for phytomedicinal drug (Ref. 11).

In human and rodent studies, it has been shown that resveratrol is well absorbed, distributed to various organs and mainly metabolised to glucuronide conjugates but has quite poor oral bioavailability and 75% of resveratrol is excreted via faeces and urine (Ref. 143). After *trans*-resveratrol was administered orally to male Sprague-Dawley rats for 28 days at repeating doses of 20 mg/(kg day), body weight and other variables did not differ between rats treated with trans-resveratrol and the control group (Ref. 141). A placebo-controlled trial performed on overweight and older adults suggest that short-term resveratrol supplementation at doses of 300 and 1000 mg/day does not adversely affect blood chemistries (Ref. 144). The European Food Safety Authority (EFSA) reports that, intended the intake level of 150 mg/day for adults is within the safety margin of synthetic *trans*-resveratrol. It also notes that diarrhoea or other gastrointestinal symptoms were reported at doses of 1 g resveratrol/day or higher. The Panel remarks on a possible interaction with medicines that are mainly metabolised by CYP2C9 since the metabolite trans-resveratrol sulphate could inhibit CYP enzymes in humans (Ref. 140).

### Astaxanthin

**Figure 8.**
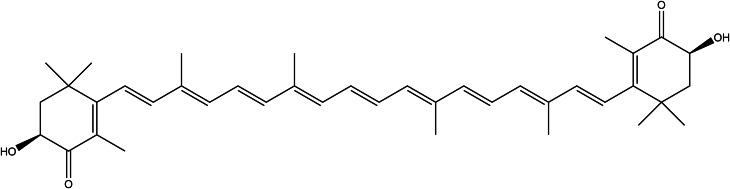
The chemical structure of astaxanthin.

Astaxanthin (AX, Fig. 8) is a carotenoid pigment found in *Haematococcus pluvialis* algae, and the marine animals that feed on them: the yeast *Pfaffia rhodozyma* (*Xanthophyllomyces dendrorhous*) and the bacterium *Paracoccus carotinifaciens* (Ref. 145).

Human nutritional sources like salmon, fish (like trout), and crustaceans provide the administration of astaxanthin in a normal diet. Astaxanthin has been found antioxidant many times higher than other antioxidant natural compounds known, especially scavenging singlet oxygen and peroxyl radicals (Refs 145, 146). Astaxanthin (AX)-containing preparations are recently popular as food supplements because of the potential to improve health in cardiovascular, neurodegenerative and skin diseases since the antioxidant activity has been affirmed (Refs 147, 148). Because of the high demand for AX supplements, the separation and production of AX from natural sources have become ecologically unsustainable. Therefore, it is synthetically available however, the compounds are not identical. Natural AX has two isomers, 3*S*,3′*S*- and 3*R*,3′*R*-stereoisomers, in free and esterified form; whereas synthetic AX consists of a mixture of the isomers (3*S*, 3′*S*)-astaxanthin, (3*R*, 3′*S*)-astaxanthin and (3*R* and 3′*R*)-astaxanthin (Ref. 147).

The studies provide information about the acute oral toxicity of natural astaxanthin, from *H. pluvialis* resulting in no adverse effect on mice. In some oral acute toxicity studies, the maximum tolerated dose of astaxanthin esters in ICR male mice was 21.5g/kg⋅BW, whereas the estimated oral LD_50_ of astaxanthin is greater than 20 g/kg⋅BW, observing no abnormalities in diet, behaviour, body weight or organ weight at mentioned a dose in pregnant mice. The repeat-dose toxicity test ranging from 100 to 500 mg/kg⋅BW astaxanthin showed no abnormalities in clinical observation throughout the pregnancy as well (Ref. 148). In a subchronic toxicity study of synthetic [3*S*, 3′*S*]-Astaxanthin in a gelatin/carbohydrate formulation, researchers determined a high dose of 700–920 mg/kg BW/day did not cause any adverse effects on Wistar rats (Ref. 149). Synthetic [3*S*, 3′*S*]-astaxanthin is nongenotoxic but rat carcinogenicity has been observed in females, at 200 mg/kg BW/day and above (Ref. 150).

Brandler and Williamson 2019 listed eight clinical studies, testing natural AX at the safety of high doses ranging from 8 to 45 mg/day and over 4 to 12 weeks. No serious adverse effects and no changes in liver parameters were observed in any of the clinical studies except a red coloration of the stool as a minor effect was noted at the beginning. According to the review, natural AX has been revealed as a clinically safe supplement at short-term daily doses of up to 100 mg and long-term daily doses averaging between 8 and 12 mg (Ref. 147).

According to the European Commission products that have AX-rich oleoresin from *H. pluvialis* should not exceed 8 mg of AX, the maximum ADI level (Ref. 151). Additionally, the commission stipulated that companies add labelling for infants, children and adolescents younger than 14 years to not be consumed. USA authorities evaluate the safety condition of AX supplements into three categories: FDA-affirmed generally recognised as safe (GRAS), self-affirmed GRAS and New Dietary Ingredient Notifications. Regarding the clinical studies on the administration of astaxanthin, authorities all over the world determine the recommended or approved doses under the category ‘novel food ingredient’ ranging between 2 and 24 mg daily (Ref. 147). Daily doses of maximum 12 mg is approved in Canada as well as Australia/New Zealand (Ref. 147).

### Curcumin

**Figure 9.**
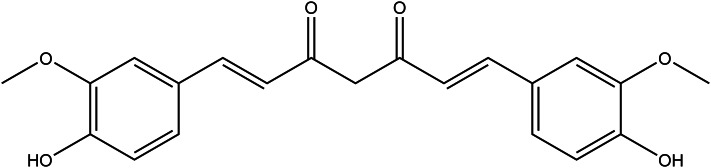
The chemical structure of curcumin.

Curcumin (1,7-bis(4-hydroxy-3-methoxyphenyl)-1,6-heptadiene-3,5-dione, Fig. 9), has been reported to exert several health effects as the main natural polyphenol found in the rhizome of *Curcuma longa* L. (turmeric). Most of the benefits of curcumin are attributed to its antioxidant and anti-inflammatory effects. It is stated that the high free radical scavenging activity and inhibitory effect on acute and chronic inflammation are due to the electron-donating group on the phenolic rings of curcumin (Ref. 152). In a study performed on human brain tumour cells, curcumin inhibited telomerase activity in all the telomerase-positive cell lines. Long-term curcumin treatment has also resulted in significant telomere shortening in cancer cell lines, which explains its potent anti-tumorigenic and antiproliferative activity (Ref. 153). Curcumin is now one of the most consumed food supplements in several countries. It is also accepted as a yellow pigment being used as a food additive (Ref. 154). In some acute toxicity studies with a single dose of 5.000 mg/kg body weight curcumin given orally to Swiss albino mice or albino Wistar rats, no toxic effects or adverse signs were observed for 14 days (Ref. 155).

Turmeric and curcumin are approved as nonmutagenic and nongenotoxic (Ref. 155). Both turmeric and curcumin showed no mutagenic activity in bacteria treated with turmeric preparations containing up to 85% curcumin. The reproductive toxicity study also concluded that the NOAEL is 250–320 mg/kg BW/day (Ref. 154). Curcumin is absorbed in rats quantitatively higher than in humans, with results varying from 3.88–60% of the administered dose (Ref. 156).

According to JECFA (The Joint United Nations and World Health Organization Expert Committee on Food Additives) and EFSA (European Food Safety Authority) reports, the 0–3 mg/kg body weight dose is the allowable daily intake (ADI) of curcumin (Ref. 157). The United States Food and Drug Administration (FDA) has announced curcumin as being GRAS (Ref. 158). Phase I clinical trials evaluated the safety profile of curcumin and it has been established that curcumin is safe given at high doses (12 g/day) over 3 months (Ref. 159). During pregnancy, curcumin was found safe in animals but there is not enough evidence of curcumin preparations for pregnant women (Ref. 155). According to pharmacokinetic interaction with drug studies, the researchers refer to the possibility of curcumin, inhibiting cytochrome (CYP) isoenzymes and P-glycoprotein so it may interact with pharmacological agents like cardiovascular drugs, antidepressants, anticoagulants, antibiotics, chemotherapeutic agents and antihistamine. More studies are needed to determine the exact potential (Ref. 160).

Curcumin exhibits poor absorption, rapid metabolism and systemic elimination, resulting in poor bioavailability (Ref. 157). Therefore, in recent studies, scientists generate different formulations and complexes by nano-formulation techniques to increase the bioavailability of curcumin and its metabolites. Those supplements should be evaluated for their safety, toxicology and pharmacokinetics (Refs 161, 162, 165).

## Conclusion and future perspective

Aging is the most prominent risk factor for many diseases, which is considered to be a complicated biological process. The biology of aging has only lately been thoroughly explored by scientists, who have focused on the evolutionarily conserved mechanisms of aging available to control functional decreases and the onset of disorders associated with aging processes. Regulatory hallmark methodologies that describe how aging is connected to physiological processes and lifestyle choices have provided a path for possible medication development.

Natural products are connected to age-associated chronic diseases, according to the combined findings from in vitro, animal, and clinical investigations over the past few decades. The body contains more plant-based nutraceuticals, such as antioxidants and anti-inflammatory compounds, when it consumes diets high in natural foods. Furthermore, plants and botanicals, medicinally derived substances from nature have been shown to have anti-aging characteristics and are utilised as a springboard for developing effective anti-aging medications. The scientific community now makes considerable use of dietary supplements and small-molecule natural products to find aging mechanisms that have been preserved throughout evolution. Additionally, they have been shown to affect senescence, nutrient-sensing metabolic signalling and mitochondrial function to slow down cellular aging and diseases associated with aging, including metabolic, cardiovascular, neurological and degenerative joint conditions.

Despite significant efforts, the most of our knowledge regarding the possible therapeutic advantages of natural products and nutritional supplements during cellular aging originates from fundamental studies employing native species that have been carefully modified for laboratory use. We cannot be assured, therefore, of the extent to which these natural products and dietary supplements alter aging mechanisms to reflect the effectiveness of aging in outbred populations of various environments. It is necessary to find solutions to the low bioavailability, off-target and steric effects problems related to natural products to produce information that could more easily be applied to human aging.

Additionally, research on the effects of natural products and dietary supplements on the health of the senior population has produced conflicting results in human clinical studies. A small sample size characterises the majority of these trials, and subgroup and sensitivity analyses are typically lacking. To identify the sequence of actual events that are crucial for comprehending the efficacy of natural products and dietary supplements in extending life, carefully controlled, large-scale interventional trials, along with biomedical informatics and multi-omics data, are required. Together, these initiatives will enable us to determine the effectiveness and discover novel genes associated with longevity that are impacted by these bioactive foods and dietary supplements.

Although natural products have benefits against aging with different molecular mechanisms, they should be used with caution due to limited information about their possible toxic properties and unknown dose interval. It is recommended that natural goods be treated with the same ‘hormesis’ theory that is used to describe the health benefits of calorie restriction and exercise. Many natural substances have a hormetic effect, such as low doses are advantageous while high doses are poisonous. Additionally, when natural products are taken in conjunction with pharmacological agents, the precipitant natural products might affect drug disposition and delivery, hence boosting or decreasing the therapeutic impact of the target drugs. Polypharmacy is another very common problem among elderly patients due to their chronic conditions. Older people also frequently use nutritional supplements and natural medications. Although, herbal medicinal products and dietary supplements are typically excluded from traditional definitions of polypharmacy, however, their possible interactions with other medications make them more likely to cause adverse drug interactions. Therefore, healthcare professionals should carefully follow elderly patients and routinely ask questions about the other medications that are not prescribed.

Even though the scientific studies implicate that most natural products are safe, tolerable and non-toxic, it is necessary to perform well-designed, large-scale randomised control trials to evaluate the short- and long-term effects and efficacy of these products. To conclusively show if natural products could extend the human lifespan, however, real-world clinical trials and fundamental research on dietary supplements and natural products are still needed.
